# Supplementary material for: CSF1R+ macrophage and osteoclast depletion impairs neural crest proliferation and craniofacial morphogenesis
Source: Development. 2026 May 7;153(16):dev205423. doi: 10.1242/dev.205423 (PMC13200734; doi:10.1242/dev.205423)
Supplement: Supplementary information [file develop-153-205423-s1.pdf]

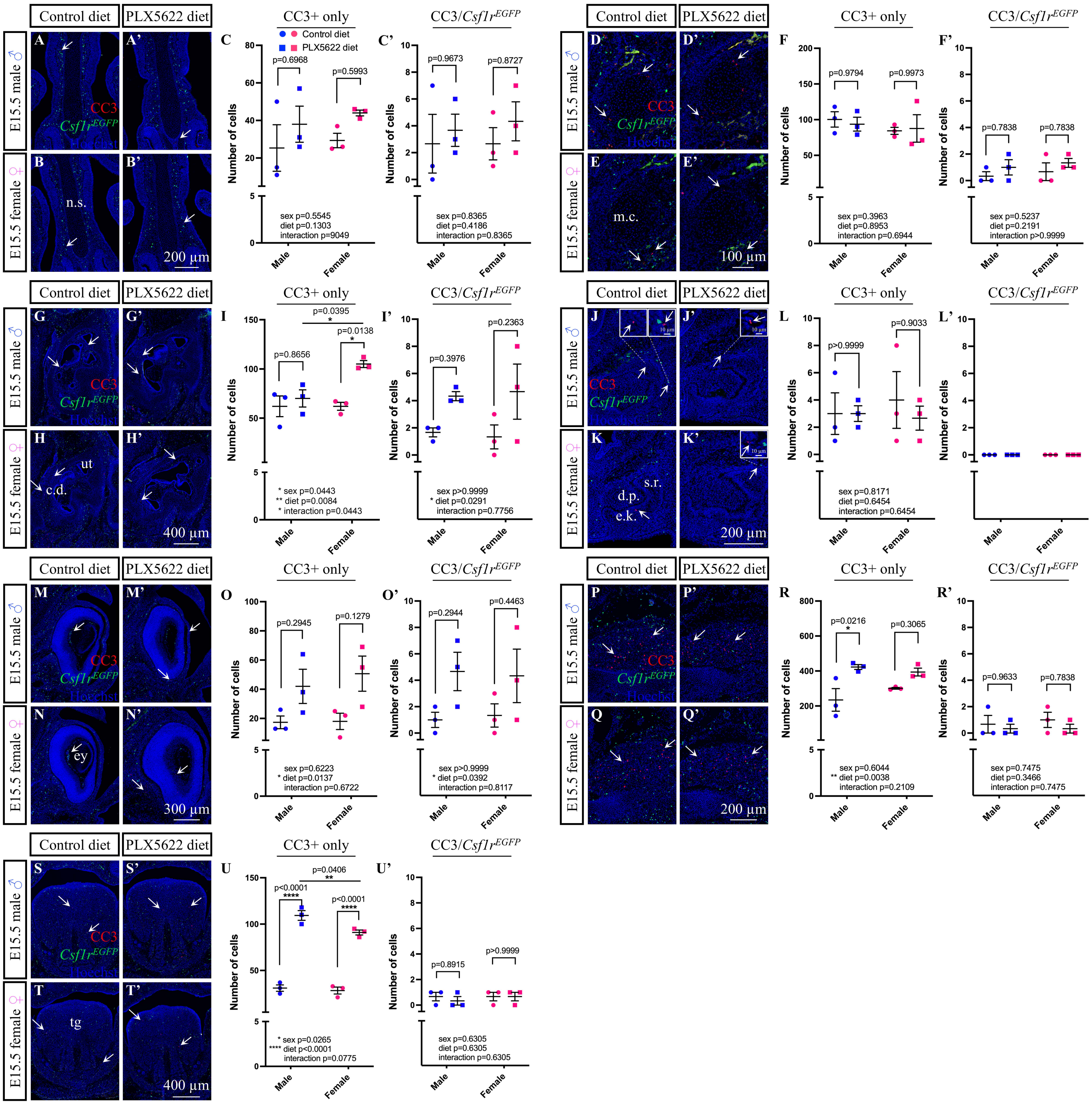

Control diet
PLX5622 diet
E15.5 male
E15.5 female
CC3
Csf1r EGFP
Hoechst
n.s.
m.c.
c.d.
ut
s.r.
d.p.
e.k.
ey
tg
200 µm
100 µm
400 µm
300 µm
10 µm
CC3+ only
CC3/Csf1r EGFP
Control diet
PLX5622 diet
Number of cells
Male
Female
p=0.6968
p=0.5993
sex p=0.5545
diet p=0.1303
interaction p=9049
p=0.9673
p=0.8727
sex p=0.8365
diet p=0.4186
interaction p=0.8365
p=0.9794
p=0.9973
sex p=0.3963
diet p=0.8953
interaction p=0.6944
p=0.7838
p=0.7838
sex p=0.5237
diet p=0.2191
interaction p>0.9999
p=0.0395
p=0.0138
p=0.8656
* sex p=0.0443
** diet p=0.0084
* interaction p=0.0443
p=0.3976
p=0.2363
sex p>0.9999
* diet p=0.0291
interaction p=0.7756
p>0.9999
p=0.9033
sex p=0.8171
diet p=0.6454
interaction p=0.6454
p=0.2945
p=0.1279
sex p=0.6223
* diet p=0.0137
interaction p=0.6722
p=0.2944
p=0.4463
sex p>0.9999
* diet p=0.0392
interaction p=0.8117
p=0.0216
p=0.3065
sex p=0.6044
** diet p=0.0038
interaction p=0.2109
p=0.9633
p=0.7838
sex p=0.7475
diet p=0.3466
interaction p=0.7475
p=0.0406
p<0.0001
p<0.0001
* sex p=0.0265
**** diet p<0.0001
interaction p=0.0775
p=0.8915
p>0.9999
sex p=0.6305
diet p=0.6305
interaction p=0.6305

Fig. S1. Exposure to PLX5622 During Gestation Leads to Tissue-Specific Increases in Apoptosis. (A-U') Immunofluorescent staining and quantification of active cleaved Caspase 3 (CC3) single-positive (arrows) and CC3+/*Csf1r*$^{EGFP+}$ double-positive cells in and around the E15.5 nasal septum (n.s.; A-C'), Meckel's cartilage (m.c.; D-F'), ear (G-I'), maxillary incisor (J-L'), eye (ey; M-O'), trigeminal (P-R'), and tongue (tg; S-U'). *n*=3 embryos per sex/treatment from two to three dams. Abbreviations: c.d., cochlear duct; d.p., dental papilla; e.k., enamel knot; s.r., stellate reticulum; ut, utricle. Counts represent mean ± SEM and were analyzed by a two-way ANOVA with Tukey's post-hoc test.

Development • Supplementary information

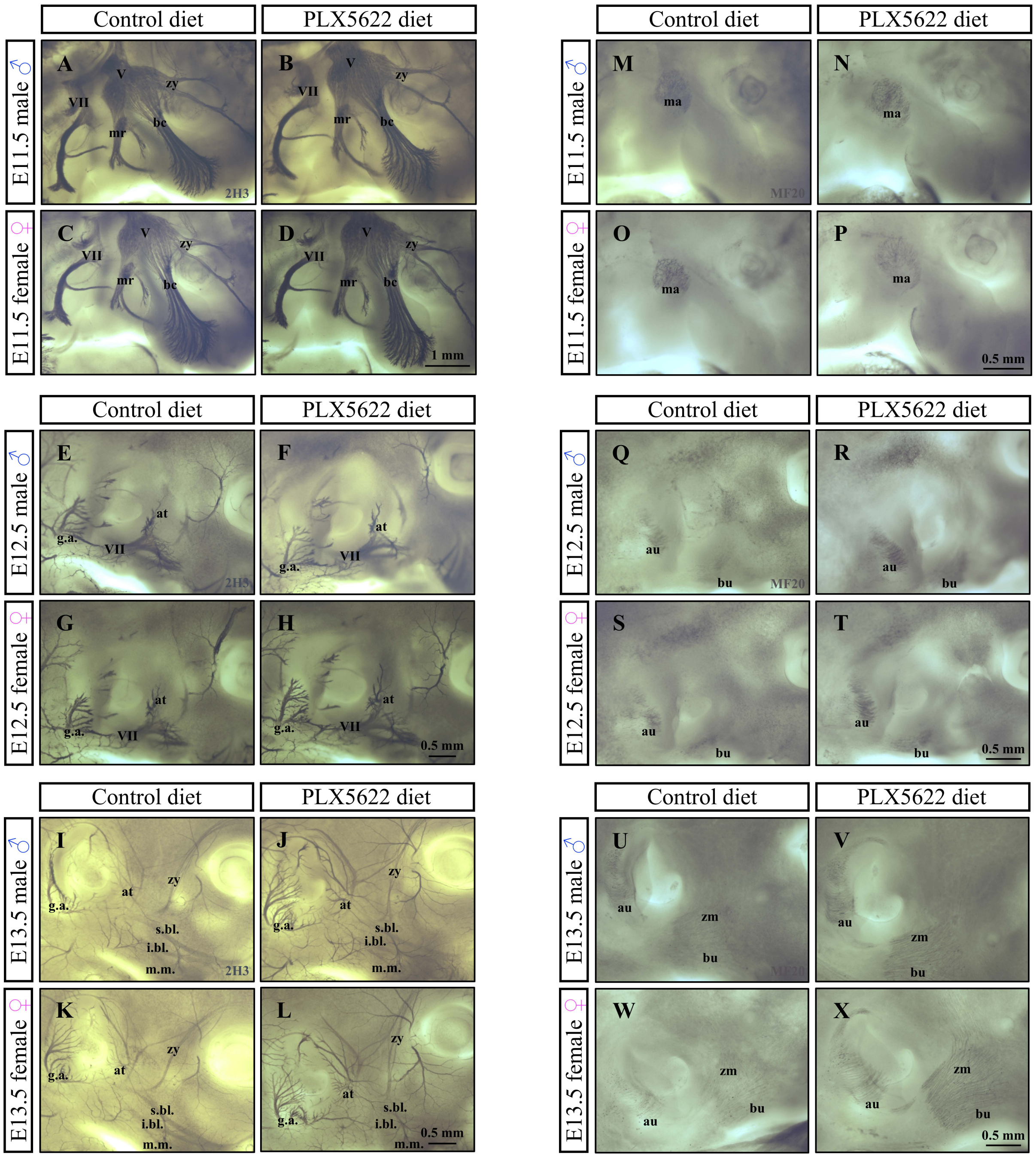

Fig. S2. Prenatal Exposure to PLX5622 Does Not Impact Nerve or Muscle Development. (A-L) Whole-mount 2H3 immunostaining of E11.5 (A-D), E12.5 (E-H), and E13.5 (I-L) CD1 embryos. *n*=3-7 embryos per sex/treatment/time-point from two to three dams. (M-X) Whole-mount MF20 immunostaining of E11.5 (M-P), E12.5 (Q-T), and E13.5 (U-X) CD1 embryos. *n*=3-4 embryos per sex/treatment/time-point from two to three dams. Abbreviations: V, trigeminal nerve; VII, facial nerve; at, auriculotemporal nerve; au, auricularis; bc, buccal branch of the trigeminal; bu, buccinator; g.a., great auricular nerve; i.bl., inferior buccolabial nerve; ma, masseter; mr, marginal branch of the trigeminal; m.m., marginal mandibular nerve; s.bl., superior buccolabial nerve; zm, zygomaticomandibularis; zy, zygomatic branch of the trigeminal.

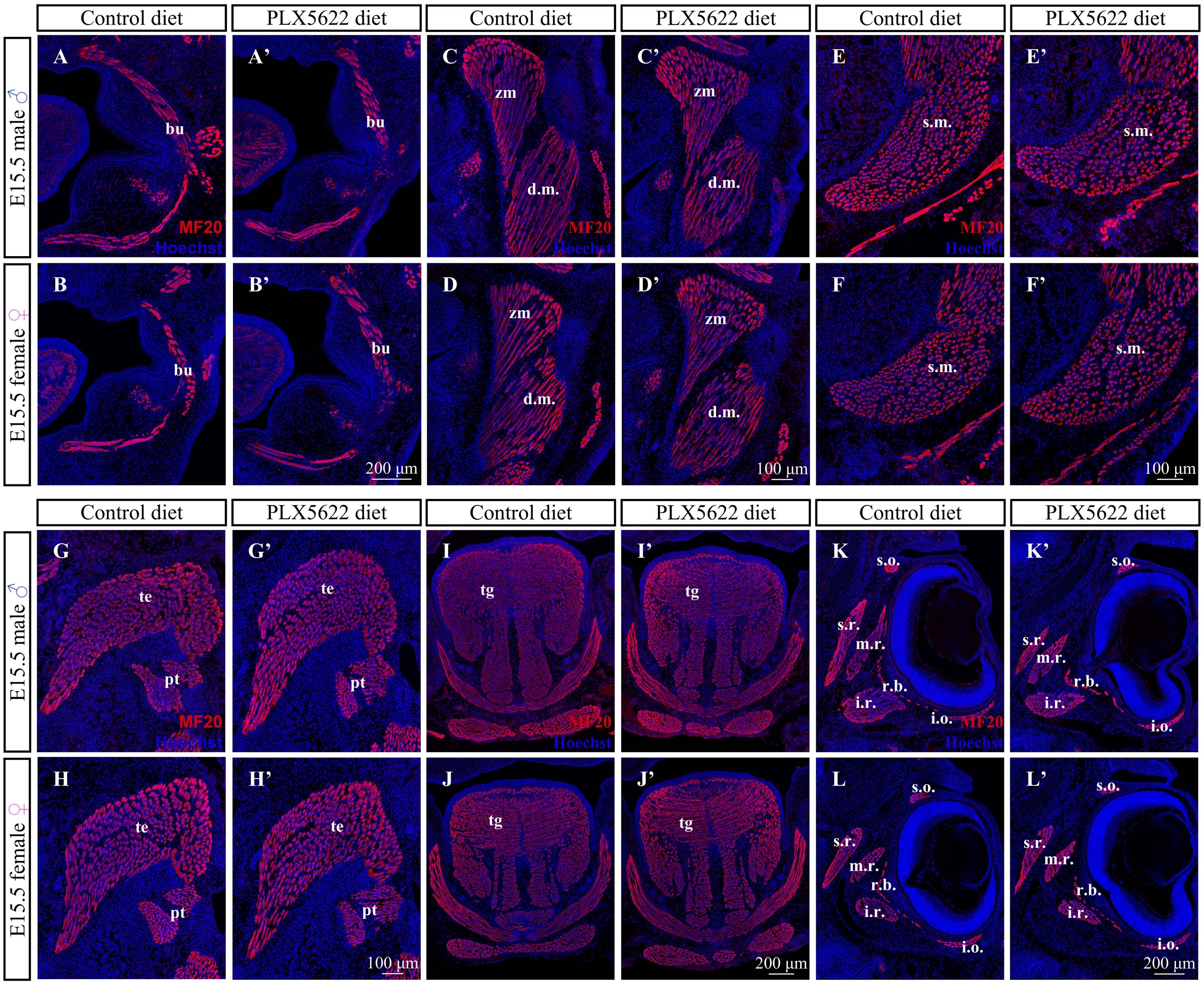

Fig. S3. Gestational Exposure to PLX5622 Does Not Disrupt Muscle Fiber Structure. (A-L’) Immunofluorescent staining with MF20 in E15.5 C57BL/6 craniofacial tissue cryosections suggest that muscle fiber diameter and cellularity are comparable between control and PLX5622 embryos in the buccinator (bu; A-B’), zygomaticomandibularis (zm; C-D’), masseter (C-F’), temporalis (te) and pterygoid (pt; G-H’), tongue (tg; I-J’), and extraocular muscles (K-L’). *n*=3 embryos per sex/treatment from two to three dams. Abbreviations: d.m., deep masseter; i.o., inferior oblique; i.r., inferior rectus; m.r., medial rectus; r.b., retractor bulbi; s.m., superficial masseter; s.o., superior oblique; s.r., superior rectus.

Development • Supplementary information

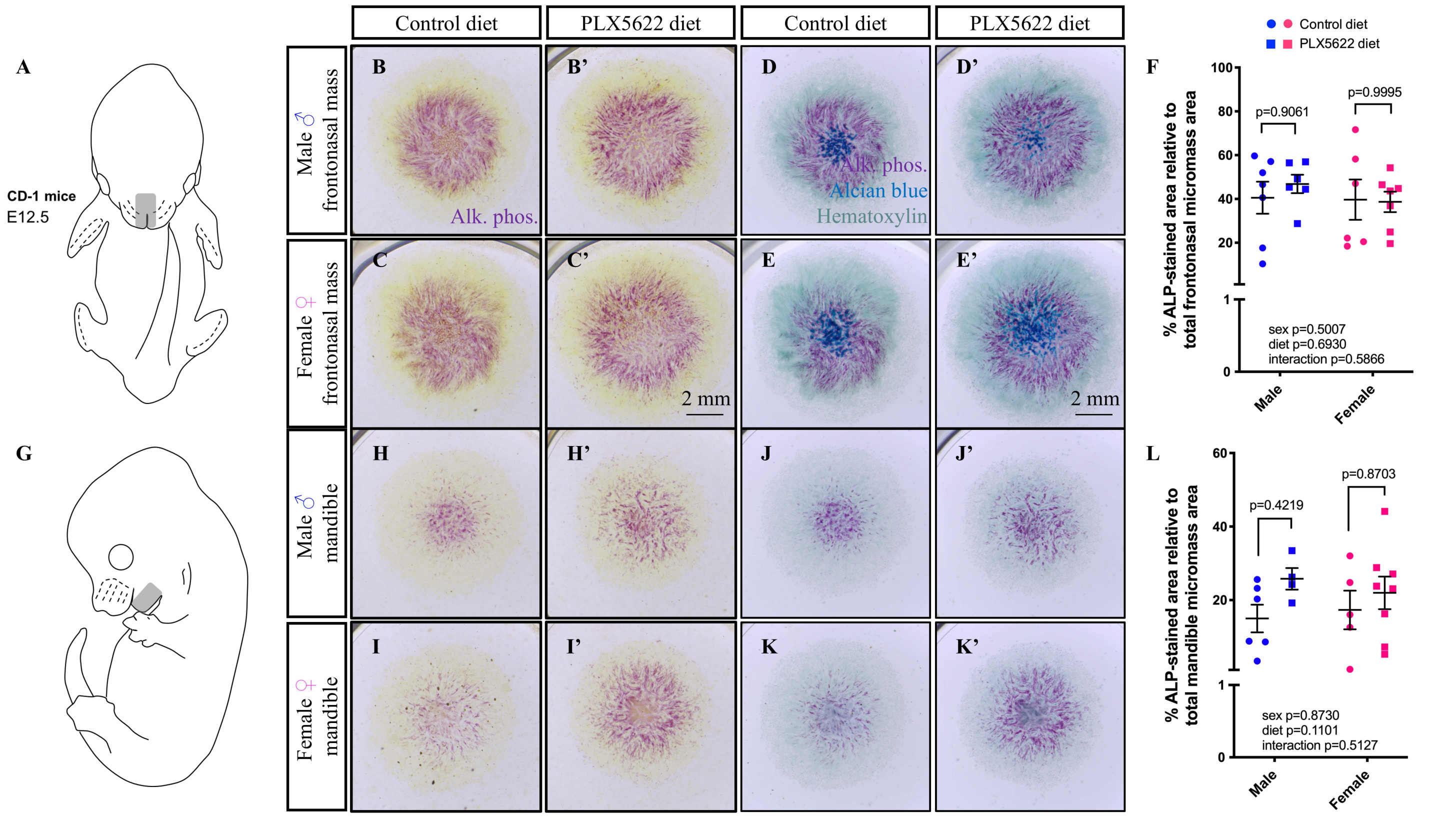

Fig. S4. Assessing Osteogenesis in Facial Micromass Cultures Collected from Embryos Exposed to PLX5622 During Gestation. (A and G) Schematic illustrating micro-dissected area for collecting mesenchymal cells from the E12.5 CD1 frontonasal mass (A) and mandible (G) for micromass culture. (B-F, H-L) Alkaline phosphatase (Alk. phos. or ALP), Alcian blue, and hematoxylin staining and quantification of E12.5 micromass cultures collected from the frontonasal mass (B-F) and mandible (H-L) of control and PLX5622 embryos. *n*=4-8 cultures per sex/treatment from three dams. Measurements represent mean ± SEM and were analyzed by a two-way ANOVA with Tukey's post-hoc test.

Development • Supplementary information

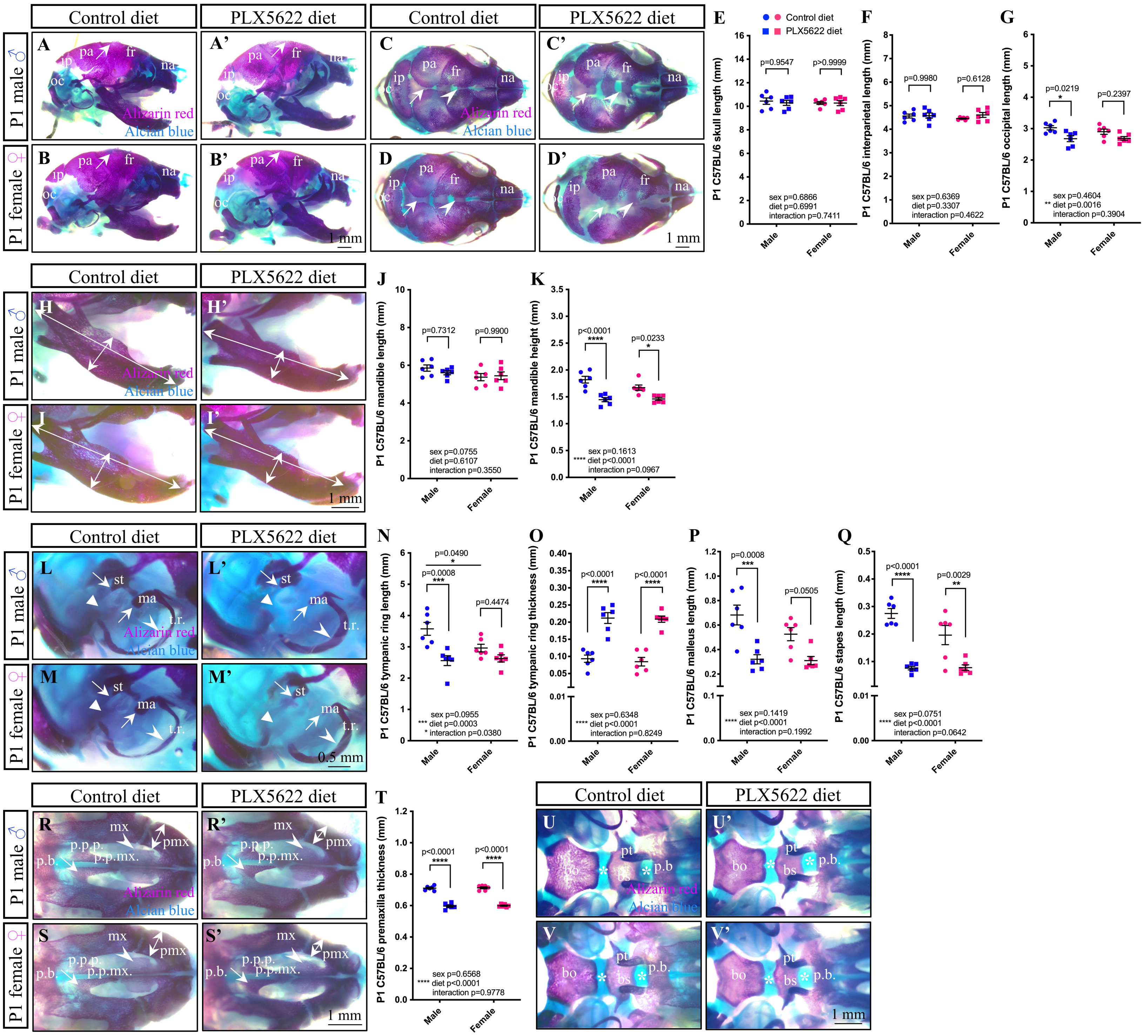

Development • Supplementary information

Fig. S5. Strain Differences in PLX5622-Driven Craniofacial Bone Morphogenesis Phenotypes. (A-B') Lateral view of P1 C57BL/6 skulls highlights doming of the cranial vault in PLX5622 pups compared to controls (arrows). (C-D') Dorsal view of P1 C57BL/6 skulls shows impaired sagittal sutures (arrows) and impaired interfrontal sutures (arrowheads) in PLX5622 pups compared to controls. (E-G) Quantification of skull (E), interparietal (F), and occipital (G) bone lengths in P1 control and PLX5622 C57BL/6 pups. (H-I') Lateral view of P1 C57BL/6 mandibles. (J-K) Quantification of mandible length (J) and height (K). Mandible length (horizontal arrows) is consistent between control and PLX5622 pups, while mandible height (vertical arrows) is disrupted in both sexes. (L-Q) Lateral view of the P1 C57BL/6 ear (L-M') and quantification of tympanic ring (t.r.) length (N) and thickness (O) and malleus (P) and stapes (Q) lengths. Arrows mark disrupted ossicle development (incus absent in C57BL/6 pups regardless of diet) of the malleus (ma), and stapes (st). Triangles mark disruptions to the otic capsule. Arrowheads mark disruptions to the tympanic ring. (R-S) Ventral view of the P1 C57BL/6 palate highlight disrupted hard palate morphogenesis (arrowheads). (T) Quantification of premaxilla (pm) thickness. (U-V') Ventral view of the P1 C57BL/6 cranial base shows widening of the spheno-occipital and intersphenoid synchondroses (asterisks) in PLX5622 pups compared to controls. *n*=6 pups per sex/treatment from three to four dams. Abbreviations: bo, basioccipital; bs, basisphenoid; fr, frontal bone; ip, interparietal bone; mx, maxilla; na, nasal bone; oc, occipital bone; pa, parietal bone; p.b., palatine bone; p.p.mx., palatine process of the maxilla; p.p.p., palatine process of the palatine; pt, pterygoid process. Measurements represent mean ± SEM and were analyzed by a two-way ANOVA with Tukey's post hoc test.

Development • Supplementary information

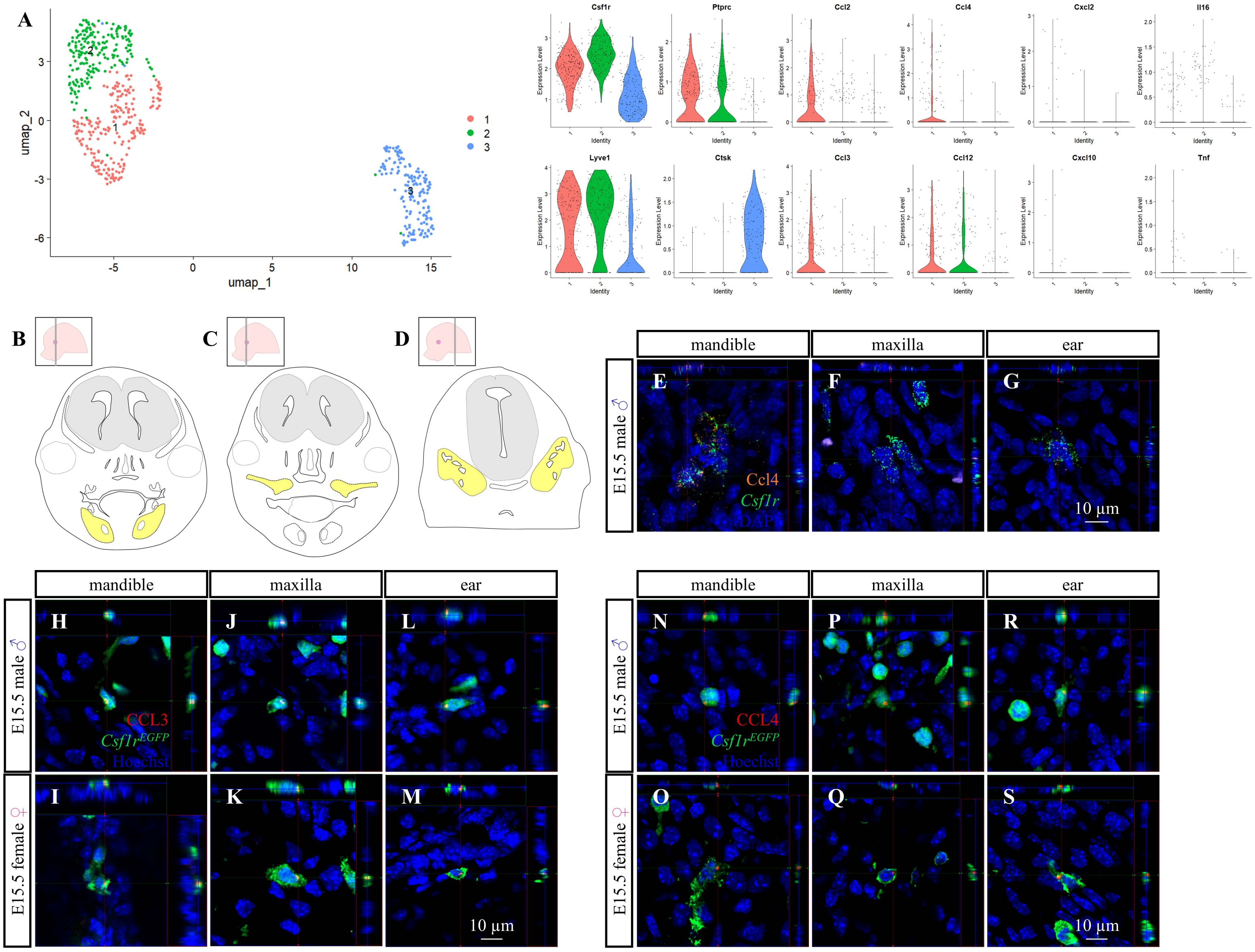

Fig. S6. Cytokines are expressed by *Csf1r*/CSF1R+ cells in Craniofacial Tissues. (A) UMAP clustering of *Csf1r*-expressing cells from published E12.5 and E13.5 FVB/NJ and C57BL/6J craniofacial mesenchyme single-cell RNA sequencing datasets[1,2]. Violin plots show expression of a number of macrophage and/or osteoclast markers across the three clusters. Cluster 1 highly expresses *Ccl2, Ccl3, Ccl4,* and *Ccl12*. *Ccl12* is also highly expressed in cluster 2. Cluster 3 expresses osteoclast marker *Ctsk*. (B-D) Schematics illustrating which areas of coronal sections of the mandible (B), maxilla (C), and ear (D) were utilized for imaging in panels E-S. (E-G) Orthographic projections of cells expressing both *Csf1r* and *Ccl4* in the E15.5 CD1 mandible (E), maxilla (F), and ear (G). (H-S) Orthographic projections of $Csf1r^{EGFP+}$ cells that co-express CCL3 (H-M) or CCL4 (N-S) in the E15.5 mandible (H, I, N, O), maxilla (J, K, P, Q), and ear (L, M, R, S).

Development • Supplementary information

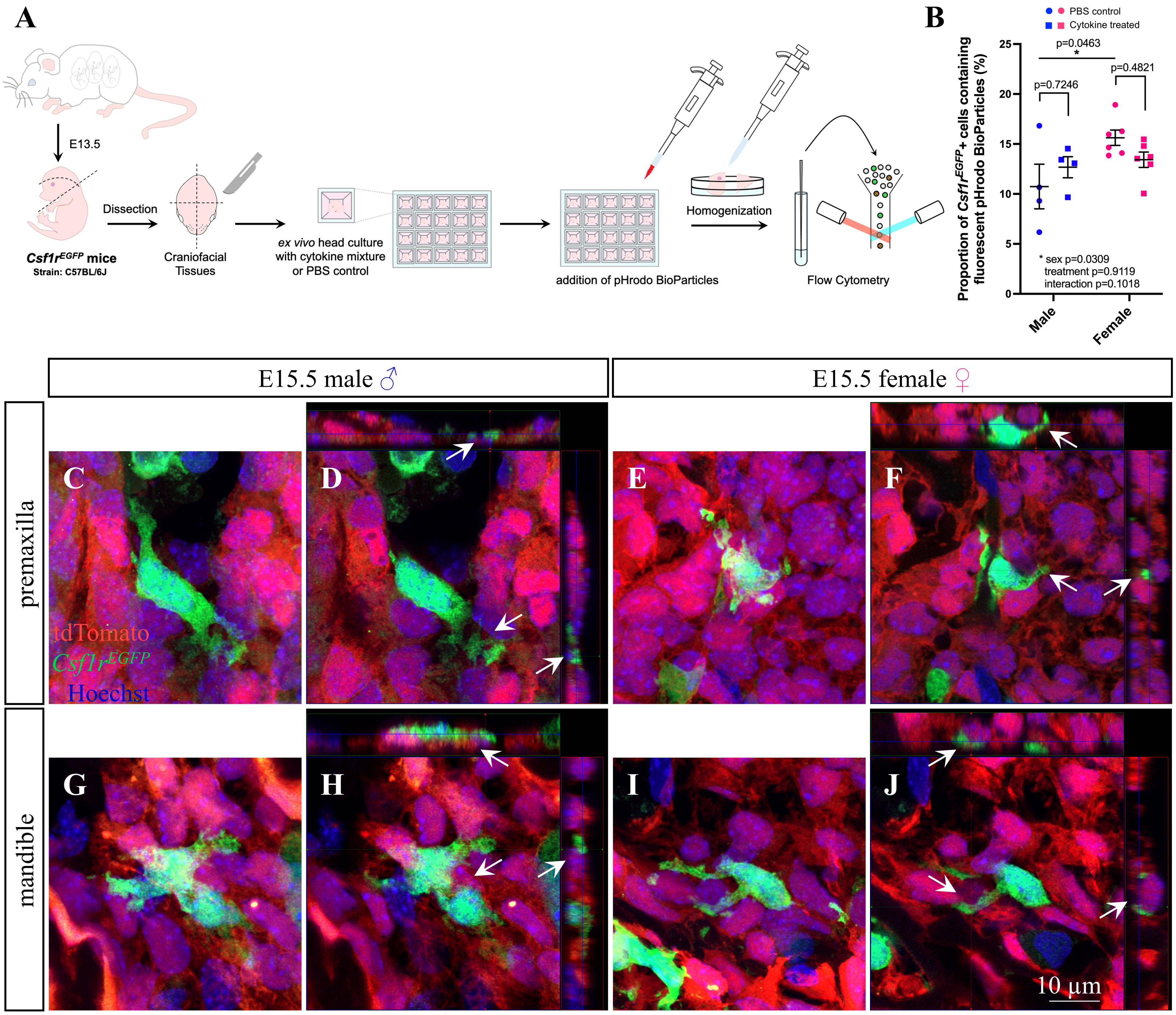

Fig. S7. Phagocytic Activity of CSF1R+ Cells is not Altered in Response to Cytokine Exposure but CSF1R+ Cells Interact with Neural Crest Cells in Craniofacial Tissues. (A) Schematic illustrating E13.5 *Csf1r*^{EGFP} craniofacial tissue collection, PBS or cytokine treatment, pHrodo *E. coli* BioParticle incubation, and flow cytometry for EGFP+ cell phagocytosis assay. (B) Quantification of the proportion of *Csf1r*$^{EGFP+}$ cells containing fluorescent pHrodo BioParticles using flow cytometry (*n*=4-6 embryos per sex/treatment from three dams). Counts represent mean ± SEM and were analyzed by a two-way ANOVA with Tukey's post-hoc test. (C-J) Immunofluorescent images (C, E, G, I) and orthographic projections (D, F, H, J) of *Csf1r*$^{EGFP+}$ cells interacting with *Wnt1*^{Cre}-driven tdTomato+ cells (arrows) in the E15.5 male (C, D, G, H) and female (E, F, I, J) premaxilla (C-F) and mandible (G-J).

Development • Supplementary information

Table S1. Exposure to PLX5622 During Gestation Does Not Affect Litter Size, Number of Embryo Resorptions or Embryo Sex Ratio.

		Number of embryos per litter	Number of embryo resorptions per litter (Number of litters with resorptions)	Sex ratio of male to female embryos per litter	Number of litters analyzed
**CD1**	**Control diet**	13.43 ± 0.69	0.43 ± 0.20 (3/7)	1.14 ± 0.25	7
	**PLX5622 diet**	11.71 ± 0.52	0.29 ± 0.18 (2/7)	1.47 ± 0.51	7
	**t-test**	p=0.0698	p=0.6110	p=0.5676	–
**C57BL/6**	**Control diet**	6.85 ± 0.56	0.62 ± 0.22 (7/26)	1.28 ± 0.20	26
	**PLX5622 diet**	7.29 ± 0.49	0.91 ± 0.29 (10/23)	1.53 ± 0.28	23
	**t-test**	p=0.5683	p=0.4176	p=0.4612	–

Table S2. Number and Proportion of *Csf1r*$^{EGFP+}$/CSF1R+ Double-Positive Cells as Compared to *Csf1r*$^{EGFP+}$ Cells in and Around Control Craniofacial Structures.

Structure	Sex	Total number of *Csf1r*$^{EGFP+}$/CSF1R+ double-positive cells	Total number of *Csf1r*$^{EGFP+}$ cells	Proportion of *Csf1r*$^{EGFP+}$/CSF1R+ double-positive cells versus all *Csf1r*$^{EGFP+}$ cells (%)
**Nasal septum**	**M (*n* = 3)**	2792	3117	89.6%
	**F (*n* = 3)**	2208	2649	83.3%
**Meckel's cartilage**	**M (*n* = 3)**	551	654	84.3%
	**F (*n* = 3)**	483	633	76.3%
**Ear**	**M (*n* = 3)**	2693	2875	93.7%
	**F (*n* = 3)**	2279	2449	93.1%
**Maxillary incisor**	**M (*n* = 3)**	19	53	35.8%
	**F (*n* = 3)**	27	67	40.3%
**Eye**	**M (*n* = 3)**	1440	1582	91.0%
	**F (*n* = 3)**	1436	1538	93.4%
**Trigeminal**	**M (*n* = 3)**	601	651	92.3%
	**F (*n* = 3)**	573	616	93.0%
**Tongue**	**M (*n* = 3)**	4073	4231	96.3%
	**F (*n* = 3)**	3329	3447	96.6%
**TOTAL**		22,504	24,562	91.6%

Development • Supplementary information

Table S3. Luminex Multiplex Analysis of Cytokines and Chemokines with Little or No Response to Gestational PLX5622 Exposure.

Cytokine/ chemokine	Sex	Control diet concentration mean ± SEM (pg/mL)	PLX5622 diet concentration mean ± SEM (pg/mL)	Control diet mean ± SEM log_2[conc (pg/mL)]	PLX5622 diet mean ± SEM log_2[conc (pg/mL)]	Two-way ANOVA results (p values) (log_2 data used) (int = interaction)	Tukey's post-hoc test p-value (CON vs. PLX)
**CCL17**	Male	0.69 ± 0.04	0.54 ± 0.01	-0.560 ± 0.088	-0.884 ± 0.036	**int: 0.4171**	***0.0146**
						**diet: ****<0.0001**	
	Female	0.78 ± 0.06	0.56 ± 0.02	-0.390 ± 0.096	-0.830 ± 0.043	**sex: 0.1258**	*****0.0006**
**CCL19**	Male	18.55 ± 1.37	21.74 ± 3.89	4.183 ± 0.103	4.308 ± 0.204	**int: 0.0654**	**0.9062**
						**diet: 0.3468**	
	Female	21.34 ± 1.68	16.27 ± 0.75	4.384 ± 0.104	4.011 ± 0.070	**sex: 0.7155**	**0.2003**
**CCL20**	Male	0.81 ± 0.03	0.70 ± 0.02	-0.318 ± 0.047	-0.523 ± 0.046	**int: 0.6653**	***0.0270**
						**diet: ****<0.0001**	
	Female	0.82 ± 0.04	0.69 ± 0.02	-0.295 ± 0.060	-0.542 ± 0.039	**sex: 0.9652**	****0.0057**
**CCL21**	Male	5542.67 ± 713.41	3811.79 ± 374.76	12.333 ± 0.197	11.831 ± 0.162	**int: *0.0146**	**0.1859**
						**diet: 0.7361**	
	Female	3868.52 ± 431.87	5058.44 ± 573.44	11.846 ± 0.163	12.231 ± 0.164	**sex: 0.8022**	**0.4008**
**CXCL9**	Male	2.29 ± 0.77	1.11 ± 0.09	0.742 ± 0.377	0.107 ± 0.120	**int: 0.3338**	**0.3070**
						**diet: **0.0015**	
	Female	2.11 ± 0.44	0.82 ± 0.06	0.819 ± 0.303	-0.315 ± 0.100	**sex: 0.5021**	***0.0174**
**Eotaxin**	Male	33.34 ± 3.83	32.13 ± 3.11	4.981 ± 0.169	4.948 ± 0.148	**int: 0.6778**	**>0.9999**
						**diet: 0.5333**	
	Female	34.89 ± 6.01	28.58 ± 1.74	4.985 ± 0.213	4.817 ± 0.084	**sex: 0.6949**	**0.9762**
**IGF-1**	Male	1182.74 ± 123.16	1769.62 ± 91.52	10.151 ± 0.139	10.774 ± 0.075	**int: 0.0633**	***0.0460**
						**diet: 0.0588**	
	Female	1365.03 ± 243.37	1239.96 ± 104.38	10.233 ± 0.254	10.239 ± 0.115	**sex: 0.1668**	**>0.9999**
**IL-2**	Male	0.85 ± 0.04	0.70 ± 0.07	-0.247 ± 0.073	-0.579 ± 0.149	**int: *0.0300**	**0.1660**
						**diet: 0.4389**	
	Female	0.74 ± 0.04	0.83 ± 0.06	-0.458 ± 0.075	-0.296 ± 0.111	**sex: 0.7438**	**0.7101**
**IL-9**	Male	17.39 ± 4.63	10.31 ± 2.58	3.781 ± 0.344	3.084 ± 0.300	**int: *0.0473**	**0.3448**
						**diet: 0.7448**	
	Female	7.28 ± 1.63	10.26 ± 1.83	2.663 ± 0.248	3.169 ± 0.266	**sex: 0.0858**	**0.6149**
**IL-16**	Male	89.24 ± 21.64	66.98 ± 10.52	6.216 ± 0.287	5.946 ± 0.202	**int: 0.3450**	**0.8433**
						**diet: *0.0417**	
	Female	92.99 ± 22.11	48.46 ± 3.61	6.282 ± 0.284	5.568 ± 0.105	**sex: 0.5060**	**0.1511**
**MMP-9**	Male	571.39 ± 76.38	463.28 ± 39.49	9.070 ± 0.172	8.810 ± 0.131	**int: 0.7126**	**0.4843**
						**diet: 0.1056**	
	Female	506.99 ± 33.99	448.62 ± 25.86	8.956 ± 0.108	8.791 ± 0.081	**sex: 0.6038**	**0.7972**
**OPN**	Male	3169.85 ± 320.26	3159.47 ± 191.52	11.579 ± 0.131	11.604 ± 0.088	**int: 0.1392**	**0.9984**
						**diet: 0.2078**	
	Female	3218.71 ± 318.29	2538.11 ± 102.01	11.601 ± 0.133	11.300 ± 0.059	**sex: 0.1967**	**0.2161**
**TIMP-1**	Male	1202.48 ± 161.46	1300.59 ± 96.37	10.141 ± 0.175	10.311 ± 0.114	**int: 0.0560**	**0.8398**
						**diet: 0.4242**	
	Female	1393.82 ± 153.51	1035.84 ± 94.67	10.376 ± 0.157	9.973 ± 0.123	**sex: 0.7222**	**0.2186**
**VEGF**	Male	4.86 ± 0.92	2.67 ± 0.82	2.031 ± 0.324	1.004 ± 0.361	**int: *0.0196**	**0.0957**
						**diet: 0.3502**	
	Female	3.58 ± 0.61	4.32 ± 0.47	1.596 ± 0.329	2.051 ± 0.141	**sex: 0.3170**	**0.7112**

CSF-1, CXCL5, EPO, GM-CSF, IFN-γ, IL-1α, IL-1β, IL-3, IL-4, IL-5, IL-7, IL-10, IL-11, IL-12p40, IL-12p70, IL-13, IL-15, IL-17, IL-20, and LIF content were at or below the level of detection.

Table S4. Comparison of Phenotypes Between PLX5622 Mice and *Csf1/Csf1r* Mutant Rodent Models.

**PLX5622 mice (gestational exposure)**	***Csf1$^{op/op}$* mice**	***Csf1r* KO mice**	***Csf1$^{tl/tl}$* rat**	***Csf1r* KO rat**	***Csf1r$^{\Delta FIRE/\Delta FIRE}$* mouse**
**CD1** (this study)[3,4]<br><br>**C57BL/6J:C57 BL/6N** (this study)	**C3FeB6F1/J *A/ A^{w-J}*** [5-14]<br><br>**C3FeB6F1/J *A/ A^{w-J}* x CD1**[15,16]<br><br>**C57BL/6J**[12]<br><br>**C57BL/6N**[17]<br><br>**FVB/NJ**[16,18-20]	**C3FeB6F1/J *A/ A^{w-J}* x CD1**[15]<br><br>**C57BL/6**[21,22]<br><br>**C57BL/6N**[17]<br><br>**FVB/NJ**[19,20,23,24]<br><br>**FVB.129X1**[25]	**Fischer**[26-35]<br><br>**Osborne-Mendel**[36]<br><br>**Lewis**[37]	**Dark Agouti**[38-42]<br><br>**Sprague Dawley**[39]<br><br>**DA:SD**[43-45]	**C57BL/6J**[46]<br><br>**C57BL/6J:CBA**[46-51]
Significant depletion of macrophages throughout craniofacial tissues (this study)[3]:<br><br>21-63% depletion in E11.5-17.5 whole head (this study)<br><br>39-73% depletion around E15.5 nasal septum (this study)<br><br>61-78% depletion around E15.5 Meckel's cartilage (this study)<br><br>65-80% depletion in E15.5 ear (this study)<br><br>57-79% depletion in E15.5 eye (this study)<br><br>41-59% depletion in	Significant reduction in most tissue macrophages (26-100%)[5,6,15,16]<br><br>50-65% reduction in ear[7]<br><br>reduction in tooth pulp, gingiva, alveolar bone, tongue[52]	Significant reduction in tissue macrophages (32-93%)[15]<br><br>~50-100% reduction in cochlea[21,22]	Significant reduction in macrophages in peritoneal and pleural lavages (>98%)[31]	Significant reduction in tissue macrophages (~30-100%)[38-40,42,45]	Normal numbers of macrophages in most tissues[47-49]<br><br>~50-100% depletion of Langerhans cells and cardiac, kidney, and large peritoneal macrophages[48]<br><br>absence of embryonic macrophages up to E13[48]

Development • Supplementary information

E15.5 trigeminal (this study)<br><br>48-73% depletion in E15.5 tongue (this study)					
~99% microglial depletion in hypothalamus by E15.5[4]	0-64% reduction in microglia[8-10,20]<br><br>24-30% microglial reduction in hypothalamus[53]<br><br>No microglial reduction in adulthood in C57BL/6N[17]	~99% reduction in microglia in hypothalamus, ~94% reduction in overall brain[17,20,25]<br><br>~85% reduction in microglia by E12.5[20]	Not shown	Absence of microglia[39,41,45]	Absence of microglia[47-51]
Absence of embryonic osteoclasts and TRAP activity at E15.5 (this study)	Reduction of osteoclasts and TRAP activity[11,12,15,16,18,29,52]<br><br>Remaining osteoclasts are small and abnormal[12,29]	Further reduction of osteoclasts and TRAP activity compared to *Csf1*$^{op/op}$ mice[15]<br><br>Absence of normal osteoclasts[23]	Absence (≥99%) of osteoclasts and TRAP activity[26-32,35]	Absence of osteoclasts and TRAP activity[38,45]	Normal numbers of osteoclasts and TRAP activity[48]
Decreased cranio-skeletal size[3]; decreased skull length (this study)	Decreased skull size and length[6,12,15,16,18]	Decreased skull size and length[15,25]	Decreased head size and length (not reported in study but observed in figures)[33,36]	Decreased skull size and length (not reported in study but observed in figures)[38,40,44,45]	No postnatal growth retardation[48]
Domed skull (this study)[3,4]	Domed skull[5-7,12,15,16,18]	Domed skull[15,25]	Not shown	Domed skull[41,45]	Not shown
Decreased calvarial bone size and density (interparietal and occipital significant only in CD1; parietal, frontal, and nasal observed) (this study)	Deformities in flat bony plates[15,16]	Deformities in flat bony plates[15]	Not shown	Decreased calvarial bone size and density[38,40,41,43]	Not shown
Impaired cranial sutures (coronal, sagittal, and interfrontal) (this study)	Not shown	Not shown	Not shown	Impaired cranial sutures[38,40,43] (coronal, sagittal, lambdoid, and interfrontal)	Not shown

Development • Supplementary information

Decreased mandible size[3]; increased mandible bone density at E15.5, decreased mandible height at P1, decreased mandible length in P1 CD1 females only (this study)	Increased mandible bone density[15]<br><br>Decreased mandible size and length, possibly decreased mandible height (not reported in study but observed in figures)[6,15,16,18]	Increased mandible bone density[15]<br><br>Decreased mandible size and length, possibly decreased mandible height (not reported in study but observed in figures)[15]	Not shown	Decreased mandible size and length (not reported in study but observed in figures)[44,45]	Not shown
Significantly smaller malleus and stapes, absence of incus (malleus not significant in C57BL/6 females; incus absent in all C57BL/6) (this study)<br><br>Shorter and thicker tympanic ring (not shorter in C57BL/6 females) (this study)<br><br>Decreased otic capsule bone density (this study)	Hypertrophy of malleus, incus, and stapes[7,13],<br><br>Increased otic capsule bone density[7] (not reported in study but observed in figures)[16]<br><br>Impaired hearing[7,14]	Impaired hearing[24]	Smaller and thicker stapes, immature bone in all auditory ossicles[34]<br><br>Majority hearing impaired[34]	Shorter tympanic ring (not reported in study but observed in figures)[44]<br><br>Increased otic capsule bone density (not reported in study but observed in figures)[44]<br><br>No evidence of sensory defects[44,45]	Not shown
Increased premaxilla bone density at E15.5 (this study)<br><br>Decreased premaxilla thickness at P1 (this study)	Increased premaxilla bone density (not reported in study but observed in figures)[15]	Increased premaxilla bone density (not reported in study but observed in figures)[15]	Not shown	Not shown	Not shown
Decreased palatine process size causing impaired palatine sutures (variable), no overt clefting (this study)	Not shown	Not shown	Not shown	Not shown	Not shown

Development • Supplementary information

Decreased cranial base bone size (only presphenoid significant) (this study)<br><br>Widened synchondroses (only intersphenoid significant) (this study)	Increased cranial base bone density (not reported in study but observed in figures)[15]	Increased cranial base bone density (not reported in study but observed in figures)[15]	Not shown	Increased cranial base bone density[44]	Not shown
Ectopic incisor enamel, extra molar cusp[4]	Toothless[5-7,12,15,16,18,54]<br><br>Unerupted molars visible with normal crowns and malformed roots[11,52]	Toothless[15,25]	Toothless[26,31,36,37]<br><br>Unerupted molars visible[36]	Toothless[38,40,41]<br><br>Unerupted rudimentary teeth visible[45]	Teeth develop and erupt normally[48]
Enlarged lateral ventricles (this study)<br><br>Lesions at cortico-striato-amygdalar boundary (this study)	Normal lateral ventricles[19]	Enlarged lateral ventricles[17,19]	Not shown	Enlarged lateral ventricles[40,41,44,45]	Normal lateral ventricles[48]<br><br>Lesions at cortico-striato-amygdalar and cortico-septal boundaries embryonically[46]

Development • Supplementary information

Table S5. Sequences of Primers Used for Genotyping for Sex and Transgenes.

REAGENT or RESOURCE	SOURCE	IDENTIFIER
Oligonucleotides		
SX (*Sly* & *Xlr*) forward primer: GATGATTTGAGTGGAAATGTGAGGTA	McFarlane et al.[55]	N/A
SX (*Sly* & *Xlr*) reverse primer: CTTATGTTTATAGGCATGCACCATGTA	McFarlane et al.[55]	N/A
GFP forward primer: AAGTTCATCTGCACCACCG	Tiscornia et al.[56]	N/A
GFP reverse primer: TCCTTGAAGAAGATGGTGCG	Tiscornia et al.[56]	N/A
Transgene forward primer for $Wnt1^{Cre}$ genotyping: CAGCGCCGCAACTATAAGAG	The Jackson Laboratory[57]	N/A
Transgene reverse primer for $Wnt1^{Cre}$ genotyping: CATCGACCGGTAATGCAG	The Jackson Laboratory[57]	N/A
Internal positive control forward primer for $Wnt1^{Cre}$ genotyping: CAAATGTTGCTTGTCTGGTG	The Jackson Laboratory[57]	N/A
Internal positive control reverse primer for $Wnt1^{Cre}$ genotyping: GTCAGTCGAGTGCACAGTTT	The Jackson Laboratory[57]	N/A
WT forward primer for $Rosa26^{tdTomato}$ genotyping: AAGGGAGCTGCAGTGGAGTA	The Jackson Laboratory[58]	N/A
WT reverse primer for $Rosa26^{tdTomato}$ genotyping: CCGAAAATCTGTGGGAAGTC	The Jackson Laboratory[58]	N/A
tdTomato forward primer for $Rosa26^{tdTomato}$ genotyping: CTGTTCCTGTACGGCATGG	The Jackson Laboratory[58]	N/A
WPRE mutant reverse primer for $Rosa26^{tdTomato}$ genotyping: GGCATTAAAGCAGCGTATCC	The Jackson Laboratory[58]	N/A

Development • Supplementary information

SUPPLEMENTAL REFERENCES

1. Angelozzi, M., Pellegrino da Silva, R., Gonzalez, M.V., and Lefebvre, V. (2022). Single-cell atlas of craniogenesis uncovers SOXC-dependent, highly proliferative, and myofibroblast-like osteodermal progenitors. Cell Rep *40*, 111045. 10.1016/j.celrep.2022.111045.
2. Rajderkar, S.S., Paraiso, K., Amaral, M.L., Kosicki, M., Cook, L.E., Darbellay, F., Spurrell, C.H., Osterwalder, M., Zhu, Y., Wu, H., et al. (2024). Dynamic enhancer landscapes in human craniofacial development. Nat Commun *15*, 2030. 10.1038/s41467-024-46396-4.
3. Nagra, A., Katsube, M., Gao, W., Rosin, J.M., and Vora, S.R. (2023). Embryonic inhibition of colony-stimulating factor 1 receptor impacts craniofacial morphogenesis. Orthod Craniofac Res *26 Suppl 1*, 20-28. 10.1111/ocr.12671.
4. Rosin, J.M., Vora, S.R., and Kurrasch, D.M. (2018). Depletion of embryonic microglia using the CSF1R inhibitor PLX5622 has adverse sex-specific effects on mice, including accelerated weight gain, hyperactivity and anxiolytic-like behaviour. Brain Behav Immun *73*, 682-697. 10.1016/j.bbi.2018.07.023.
5. Cecchini, M.G., Dominguez, M.G., Mocci, S., Wetterwald, A., Felix, R., Fleisch, H., Chisholm, O., Hofstetter, W., Pollard, J.W., and Stanley, E.R. (1994). Role of colony stimulating factor-1 in the establishment and regulation of tissue macrophages during postnatal development of the mouse. Development *120*, 1357-1372. 10.1242/dev.120.6.1357.
6. Ryan, G.R., Dai, X.M., Dominguez, M.G., Tong, W., Chuan, F., Chisholm, O., Russell, R.G., Pollard, J.W., and Stanley, E.R. (2001). Rescue of the colony-stimulating factor 1 (CSF-1)-nullizygous mouse (Csf1(op)/Csf1(op)) phenotype with a CSF-1 transgene and identification of sites of local CSF-1 synthesis. Blood *98*, 74-84. 10.1182/blood.v98.1.74.
7. Okano, T., and Kishimoto, I. (2019). Csf1 Signaling Regulates Maintenance of Resident Macrophages and Bone Formation in the Mouse Cochlea. Frontiers in Neurology *10*. 10.3389/fneur.2019.01244.
8. Kondo, Y., and Duncan, I.D. (2009). Selective reduction in microglia density and function in the white matter of colony-stimulating factor-1-deficient mice. J Neurosci Res *87*, 2686-2695. 10.1002/jnr.22096.
9. Sasaki, A., Yokoo, H., Naito, M., Kaizu, C., Shultz, L.D., and Nakazato, Y. (2000). Effects of macrophage-colony-stimulating factor deficiency on the maturation of microglia and brain macrophages and on their expression of scavenger receptor. Neuropathology *20*, 134-142. 10.1046/j.1440-1789.2000.00286.x.
10. Wegiel, J., Wisniewski, H.M., Dziewiatkowski, J., Tarnawski, M., Kozielski, R., Trenkner, E., and Wiktor-Jedrzejczak, W. (1998). Reduced number and altered morphology of microglial cells in colony stimulating factor-1-deficient osteopetrotic op/op mice. Brain Res *804*, 135-139. 10.1016/s0006-8993(98)00618-0.
11. Kodama, H., Yamasaki, A., Nose, M., Niida, S., Ohgame, Y., Abe, M., Kumegawa, M., and Suda, T. (1991). Congenital osteoclast deficiency in osteopetrotic (op/op) mice is cured by injections of macrophage colony-stimulating factor. J Exp Med *173*, 269-272. 10.1084/jem.173.1.269.
12. Marks, S.C., Jr., and Lane, P.W. (1976). Osteopetrosis, a new recessive skeletal mutation on chromosome 12 of the mouse. J Hered *67*, 11-18. 10.1093/oxfordjournals.jhered.a108657.

13. Kanzaki, S., Takada, Y., Niida, S., Takeda, Y., Udagawa, N., Ogawa, K., Nango, N., Momose, A., and Matsuo, K. (2011). Impaired vibration of auditory ossicles in osteopetrotic mice. Am J Pathol *178*, 1270-1278. 10.1016/j.ajpath.2010.11.063.
14. Michaelson, M.D., Bieri, P.L., Mehler, M.F., Xu, H., Arezzo, J.C., Pollard, J.W., and Kessler, J.A. (1996). CSF-1 deficiency in mice results in abnormal brain development. Development *122*, 2661-2672. 10.1242/dev.122.9.2661.
15. Dai, X.M., Ryan, G.R., Hapel, A.J., Dominguez, M.G., Russell, R.G., Kapp, S., Sylvestre, V., and Stanley, E.R. (2002). Targeted disruption of the mouse colony-stimulating factor 1 receptor gene results in osteopetrosis, mononuclear phagocyte deficiency, increased primitive progenitor cell frequencies, and reproductive defects. Blood *99*, 111-120. 10.1182/blood.v99.1.111.
16. Dai, X.M., Zong, X.H., Sylvestre, V., and Stanley, E.R. (2004). Incomplete restoration of colony-stimulating factor 1 (CSF-1) function in CSF-1-deficient Csf1op/Csf1op mice by transgenic expression of cell surface CSF-1. Blood *103*, 1114-1123. 10.1182/blood-2003-08-2739.
17. Erblich, B., Zhu, L., Etgen, A.M., Dobrenis, K., and Pollard, J.W. (2011). Absence of colony stimulation factor-1 receptor results in loss of microglia, disrupted brain development and olfactory deficits. PLoS One *6*, e26317. 10.1371/journal.pone.0026317.
18. Wei, S., Nandi, S., Chitu, V., Yeung, Y.G., Yu, W., Huang, M., Williams, L.T., Lin, H., and Stanley, E.R. (2010). Functional overlap but differential expression of CSF-1 and IL-34 in their CSF-1 receptor-mediated regulation of myeloid cells. J Leukoc Biol *88*, 495-505. 10.1189/jlb.1209822.
19. Nandi, S., Gokhan, S., Dai, X.M., Wei, S., Enikolopov, G., Lin, H., Mehler, M.F., and Stanley, E.R. (2012). The CSF-1 receptor ligands IL-34 and CSF-1 exhibit distinct developmental brain expression patterns and regulate neural progenitor cell maintenance and maturation. Dev Biol *367*, 100-113. 10.1016/j.ydbio.2012.03.026.
20. Ginhoux, F., Greter, M., Leboeuf, M., Nandi, S., See, P., Gokhan, S., Mehler, M.F., Conway, S.J., Ng, L.G., Stanley, E.R., et al. (2010). Fate mapping analysis reveals that adult microglia derive from primitive macrophages. Science *330*, 841-845. 10.1126/science.1194637.
21. Miwa, T., Rengasamy, G., Liu, Z., Ginhoux, F., and Okano, T. (2024). Contribution of circulating monocytes in maintaining homeostasis of resident macrophages in postnatal and young adult mouse cochlea. Sci Rep *14*, 62. 10.1038/s41598-023-50634-y.
22. Kishimoto, I., Okano, T., Nishimura, K., Motohashi, T., and Omori, K. (2019). Early Development of Resident Macrophages in the Mouse Cochlea Depends on Yolk Sac Hematopoiesis. Front Neurol *10*, 1115. 10.3389/fneur.2019.01115.
23. Dai, X.-M., Zong, X.-H., Akhter, M.P., and Stanley, E.R. (2004). Osteoclast Deficiency Results in Disorganized Matrix, Reduced Mineralization, and Abnormal Osteoblast Behavior in Developing Bone. Journal of Bone and Mineral Research *19*, 1441-1451. https://doi.org/10.1359/JBMR.040514.
24. Chitu, V., Gokhan, Ş., Nandi, S., Mehler, M.F., and Stanley, E.R. (2016). Emerging Roles for CSF-1 Receptor and its Ligands in the Nervous System. Trends in Neurosciences *39*, 378-393. https://doi.org/10.1016/j.tins.2016.03.005.
25. Bennett, F.C., Bennett, M.L., Yaqoob, F., Mulinyawe, S.B., Grant, G.A., Hayden Gephart, M., Plowey, E.D., and Barres, B.A. (2018). A Combination of Ontogeny and CNS Environment Establishes Microglial Identity. Neuron *98*, 1170-1183 e1178. 10.1016/j.neuron.2018.05.014.

26. Van Wesenbeeck, L., Odgren, P.R., MacKay, C.A., D'Angelo, M., Safadi, F.F., Popoff, S.N., Van Hul, W., and Marks, S.C., Jr. (2002). The osteopetrotic mutation toothless (tl) is a loss-of-function frameshift mutation in the rat Csf1 gene: Evidence of a crucial role for CSF-1 in osteoclastogenesis and endochondral ossification. Proc Natl Acad Sci U S A *99*, 14303-14308. 10.1073/pnas.202332999.
27. Odgren, P.R., Kim, N., van Wesenbeeck, L., MacKay, C., Mason-Savas, A., Safadi, F.F., Popoff, S.N., Lengner, C., van-Hul, W., Choi, Y., and Marks, S.C., Jr. (2001). Evidence that the rat osteopetrotic mutation toothless (tl) is not in the TNFSF11 (TRANCE, RANKL, ODF, OPGL) gene. Int J Dev Biol *45*, 853-859.
28. Marks, S.C., Jr., Mackay, C.A., Jackson, M.E., Larson, E.K., Cielinski, M.J., Stanley, E.R., and Aukerman, S.L. (1993). The skeletal effects of colony-stimulating factor-1 in toothless (osteopetrotic) rats: persistent metaphyseal sclerosis and the failure to restore subepiphyseal osteoclasts. Bone *14*, 675-680. 10.1016/8756-3282(93)90091-n.
29. Marks, S.C., Jr., Iizuka, T., MacKay, C.A., Mason-Savas, A., and Cielinski, M.J. (1997). The effects of colony-stimulating factor-1 on the number and ultrastructure of osteoclasts in toothless (tl) rats and osteopetrotic (op) mice. Tissue Cell *29*, 589-595. 10.1016/s0040-8166(97)80059-6.
30. Joseph, B.K., Marks, S.C., Jr., Hume, D.A., Waters, M.J., and Symons, A.L. (1999). Insulin-like growth factor-I (IGF-I) and IGF-I receptor (IGF-IR) immunoreactivity in normal and osteopetrotic (toothless, tl/tl) rat tibia. Growth Factors *16*, 279-291. 10.3109/08977199909069146.
31. Marks, S.C., Jr., Wojtowicz, A., Szperl, M., Urbanowska, E., MacKay, C.A., Wiktor-Jedrzejczak, W., Stanley, E.R., and Aukerman, S.L. (1992). Administration of colony stimulating factor-1 corrects some macrophage, dental, and skeletal defects in an osteopetrotic mutation (toothless, tl) in the rat. Bone *13*, 89-93. 10.1016/8756-3282(92)90365-4.
32. Yang, M., Mailhot, G., MacKay, C.A., Mason-Savas, A., Aubin, J., and Odgren, P.R. (2006). Chemokine and chemokine receptor expression during colony stimulating factor-1-induced osteoclast differentiation in the toothless osteopetrotic rat: a key role for CCL9 (MIP-1gamma) in osteoclastogenesis in vivo and in vitro. Blood *107*, 2262-2270. 10.1182/blood-2005-08-3365.
33. Marks, S.C., Lundmark, C., Wurtz, T., Odgren, P.R., MacKay, C.A., Mason-Savas, A., and Popoff, S.N. (1999). Facial development and type III collagen RNA expression: Concurrent repression in the osteopetrotic(Toothless, tl) rat and rescue after treatment with colony-stimulating factor-1. Developmental Dynamics *215*, 117-125. 10.1002/(sici)1097-0177(199906)215:2<117::Aid-dvdy4>3.0.Co;2-d.
34. Aharinejad, S., Grossschmidt, K., Franz, P., Streicher, J., Nourani, F., MacKay, C.A., Firbas, W., Plenk, H., Jr., and Marks, S.C., Jr. (1999). Auditory ossicle abnormalities and hearing loss in the toothless (osteopetrotic) mutation in the rat and their improvement after treatment with colony-stimulating factor-1. J Bone Miner Res *14*, 415-423. 10.1359/jbmr.1999.14.3.415.
35. Wisner-Lynch, L.A., Shalhoub, V., and Marks, S.C., Jr. (1995). Administration of colony stimulating factor-1 to toothless osteopetrotic rats normalizes osteoblast, but not osteoclast, gene expression. Bone *16*, 611-618. 10.1016/8756-3282(95)00114-s.
36. Cotton, W.R., and Gaines, J.F. (1974). Unerupted dentition secondary to congenital osteopetrosis in the Osborne-Mendel rat. Proc Soc Exp Biol Med *146*, 554-561. 10.3181/00379727-146-38146.

37. Dobbins, D.E., Sood, R., Hashiramoto, A., Hansen, C.T., Wilder, R.L., and Remmers, E.F. (2002). Mutation of macrophage colony stimulating factor (Csf1) causes osteopetrosis in the tl rat. Biochem Biophys Res Commun *294*, 1114-1120. 10.1016/S0006-291X(02)00598-3.
38. Batoon, L., Keshvari, S., Irvine, K.M., Ho, E., Caruso, M., Patkar, O.L., Sehgal, A., Millard, S.M., Hume, D.A., and Pettit, A.R. (2024). Relative contributions of osteal macrophages and osteoclasts to postnatal bone development in CSF1R-deficient rats and phenotype rescue following wild-type bone marrow cell transfer. J Leukoc Biol *116*, 753-765. 10.1093/jleuko/qiae077.
39. Hume, D.A., Teakle, N., Keshvari, S., and Irvine, K.M. (2023). Macrophage deficiency in CSF1R-knockout rat embryos does not compromise placental or embryo development. J Leukoc Biol *114*, 421-433. 10.1093/jleuko/qiad052.
40. Keshvari, S., Caruso, M., Teakle, N., Batoon, L., Sehgal, A., Patkar, O.L., Ferrari-Cestari, M., Snell, C.E., Chen, C., Stevenson, A., et al. (2021). CSF1R-dependent macrophages control postnatal somatic growth and organ maturation. PLoS Genet *17*, e1009605. 10.1371/journal.pgen.1009605.
41. Patkar, O.L., Caruso, M., Teakle, N., Keshvari, S., Bush, S.J., Pridans, C., Belmer, A., Summers, K.M., Irvine, K.M., and Hume, D.A. (2021). Analysis of homozygous and heterozygous Csf1r knockout in the rat as a model for understanding microglial function in brain development and the impacts of human CSF1R mutations. Neurobiol Dis *151*, 105268. 10.1016/j.nbd.2021.105268.
42. Carter-Cusack, D., Huang, S., Keshvari, S., Patkar, O., Sehgal, A., Allavena, R., Byrne, R.A.J., Morgan, B.P., Bush, S.J., Summers, K.M., et al. (2025). Wild-type bone marrow cells repopulate tissue resident macrophages and reverse the impacts of homozygous CSF1R mutation. PLoS Genet *21*, e1011525. 10.1371/journal.pgen.1011525.
43. Hume, D.A., Batoon, L., Sehgal, A., Keshvari, S., and Irvine, K.M. (2022). CSF1R as a Therapeutic Target in Bone Diseases: Obvious but Not so Simple. Curr Osteoporos Rep *20*, 516-531. 10.1007/s11914-022-00757-4.
44. Hume, D.A., Caruso, M., Ferrari-Cestari, M., Summers, K.M., Pridans, C., and Irvine, K.M. (2020). Phenotypic impacts of CSF1R deficiencies in humans and model organisms. Journal of Leukocyte Biology *107*, 205-219. https://doi.org/10.1002/JLB.MR0519-143R.
45. Pridans, C., Raper, A., Davis, G.M., Alves, J., Sauter, K.A., Lefevre, L., Regan, T., Meek, S., Sutherland, L., Thomson, A.J., et al. (2018). Pleiotropic Impacts of Macrophage and Microglial Deficiency on Development in Rats with Targeted Mutation of the Csf1r Locus. J Immunol *201*, 2683-2699. 10.4049/jimmunol.1701783.
46. Lawrence, A.R., Canzi, A., Bridlance, C., Olivie, N., Lansonneur, C., Catale, C., Pizzamiglio, L., Kloeckner, B., Silvin, A., Munro, D.A.D., et al. (2024). Microglia maintain structural integrity during fetal brain morphogenesis. Cell *187*, 962-980 e919. 10.1016/j.cell.2024.01.012.
47. McNamara, N.B., Munro, D.A.D., Bestard-Cuche, N., Uyeda, A., Bogie, J.F.J., Hoffmann, A., Holloway, R.K., Molina-Gonzalez, I., Askew, K.E., Mitchell, S., et al. (2023). Microglia regulate central nervous system myelin growth and integrity. Nature *613*, 120-129. 10.1038/s41586-022-05534-y.
48. Rojo, R., Raper, A., Ozdemir, D.D., Lefevre, L., Grabert, K., Wollscheid-Lengeling, E., Bradford, B., Caruso, M., Gazova, I., Sanchez, A., et al. (2019). Deletion of a Csf1r enhancer selectively impacts CSF1R expression and development of tissue macrophage populations. Nat Commun *10*, 3215. 10.1038/s41467-019-11053-8.

49. Munro, D.A.D., Bradford, B.M., Mariani, S.A., Hampton, D.W., Vink, C.S., Chandran, S., Hume, D.A., Pridans, C., and Priller, J. (2020). CNS macrophages differentially rely on an intronic Csf1r enhancer for their development. Development *147*. 10.1242/dev.194449.
50. Kiani Shabestari, S., Morabito, S., Danhash, E.P., McQuade, A., Sanchez, J.R., Miyoshi, E., Chadarevian, J.P., Claes, C., Coburn, M.A., Hasselmann, J., et al. (2022). Absence of microglia promotes diverse pathologies and early lethality in Alzheimer's disease mice. Cell Rep *39*, 110961. 10.1016/j.celrep.2022.110961.
51. Munro, D.A.D., Bestard-Cuche, N., McQuaid, C., Chagnot, A., Shabestari, S.K., Chadarevian, J.P., Maheshwari, U., Szymkowiak, S., Morris, K., Mohammad, M., et al. (2024). Microglia protect against age-associated brain pathologies. Neuron *112*, 2732-2748.e2738. 10.1016/j.neuron.2024.05.018.
52. Nagahama, S.I., Cunningham, M.L., Lee, M.Y., and Byers, M.R. (1998). Normal development of dental innervation and nerve/tissue interactions in the colony-stimulating factor-1 deficient osteopetrotic mouse. Developmental Dynamics *211*, 52-59. 10.1002/(sici)1097-0177(199801)211:1<52::Aid-aja5>3.0.Co;2-6.
53. Cohen, P.E., Zhu, L., Nishimura, K., and Pollard, J.W. (2002). Colony-stimulating factor 1 regulation of neuroendocrine pathways that control gonadal function in mice. Endocrinology *143*, 1413-1422. 10.1210/endo.143.4.8754.
54. Pollard, J.W., and Hennighausen, L. (1994). Colony stimulating factor 1 is required for mammary gland development during pregnancy. Proc Natl Acad Sci U S A *91*, 9312-9316. 10.1073/pnas.91.20.9312.
55. McFarlane, L., Truong, V., Palmer, J.S., and Wilhelm, D. (2013). Novel PCR assay for determining the genetic sex of mice. Sex Dev *7*, 207-211. 10.1159/000348677.
56. Tiscornia, G., Singer, O., Ikawa, M., and Verma, I.M. (2003). A general method for gene knockdown in mice by using lentiviral vectors expressing small interfering RNA. Proc Natl Acad Sci U S A *100*, 1844-1848. 10.1073/pnas.0437912100.
57. The Jackson Laboratory. Protocol 25394: Standard PCR Assay - Tg(Wnt1-cre). https://www.jax.org/Protocol?stockNumber=022501&protocolID=25394.
58. The Jackson Laboratory. Protocol 29436: Standard PCR Assay - Gt(ROSA)26Sor(tdTomato-WPRE). https://www.jax.org/Protocol?stockNumber=007914&protocolID=29436.
